# Spatial variations and multilevel mixed effect analysis on determinants factors of modern contraceptive utilization among reproductive age women in Ethiopia; proven by Ethiopian mini demographic health survey 2019

**DOI:** 10.1186/s12905-022-02030-3

**Published:** 2023-02-22

**Authors:** Gosa Mankelkl, Beletu Kinfe

**Affiliations:** 1 College of Medicine and Health Science, Mizan-Tepi University, Mizan Teferi, Ethiopia; 2 College of Medicine and Health Science, Wollo University, Dessie, Ethiopia

**Keywords:** Modern contraceptive use, Multilevel, Spatial, Ethiopia

## Abstract

**Introduction:**

Globally, in 2019, there are 1.9 billion women of reproductive age (15–49), of which 1.1 billion have a need for family planning. Of these, 842 million use contraceptives, and 270 million still have an unmet need for contraception. Ethiopia is a low-income country with inadequate access to family planning (FP), especially in the developing regions. The Ethiopian government was striving to increase the number of health facility in order to provide quality maternal care and services. Increasing the modern contraceptive prevalence rate is one of the goals of the government to reduce maternal and child mortality and morbidity.

**Methods:**

Secondary data analysis was conducted using data from mini-EDHS of 2019 demographic and health Survey datasets. The study comprised a total of 8885 reproductive-age women. Spatial variations and multilevel mixed effect analysis on determinants factors of modern contraceptive use among reproductive age women in Ethiopia; evidenced by mini-EDHS 2019. Finally, the percentage and odd ratio, its 95% confidence intervals, and the result of spatial analysis were reported.

**Result:**

This study includes a total weighted sample of 8885 reproductive-age women from the 2019 mini-Ethiopian demographic and health survey. The prevalence of modern contraceptive use was 25.5% in Ethiopia. living in urban area [AOR = 2.13; 95% CI = (1.75, 2.61); *P* = 0.000], being married [AOR = 1.42; 95% CI = (1.19, 1.70); P = 0.000] were found positively associated with contraceptive use. In contrast to this, attending primary education [AOR = 0.91; 95% CI = (0.74, 1.12); *P* = 0.000]., being Muslim [AOR = 0.25; 95% CI = (0.22, 0.29); P = 0.000], being poorest [AOR = 0.54; 95% CI = (0.45, 0.66); P = 0.000] were found negatively associated with contraceptive use.

**Conclusion:**

In this study Individual and community level factors were associated with modern contraceptive use and also there were spatial variations in modern contraceptive use across the region among reproductive-age women. Empowering women to have better educational status, improving the wealth index, promoting marriage, creating awareness among rural residences women and promoting education about modern contraceptives through religiously acceptable persons, and promoting modern contraceptive use in developing regions were the key factors to improve modern contraceptive use among reproductive age women in Ethiopia.

## Introduction

The purposeful avoidance of pregnancy by the use of different techniques, sexual practices, drugs, medications, or surgical procedures is known as contraception [1]. Contraceptive methods are usually divided into modern and traditional categories. Traditional methods of contraception include the lactational amenorrhoea method, the rhythm method (periodic abstinence), withdrawal (coitus interruptus), and folk methods [2]. A modern contraceptive method is a drug or medical treatment that prevents sexual activity from leading to pregnancy [3]. modern contraceptive methods include barrier methods such as male and female condoms, diaphragm, cervical cap and sponge; hormonal contraceptives that include oral, injectable, transdermal, vaginal ring, and implants; intrauterine device (IUD) [4]. The majority of contraceptives used globally are modern methods. A modern method of family planning was used by 58% of married or in-union women of reproductive age worldwide in 2017, accounting for 92% of all contraceptive users [5]. According to the Ethiopian Mini Demographic and Health Survey of 2019 report, currently married women’s use of modern contraceptives has continuously risen from 2005, from 14 to 41% [6].

There are a variety of benefits to contraceptives, including those for women’s empowerment, maternal and child health, economic growth, and education [7]. Additionally, the modern methods of contraceptive are crucial for reducing unintended pregnancies, delaying births, and improving neonatal and child survival rates since there will be more time for good parenting and child care [8]. Several literature demonstrated that the use of modern contraceptives is influenced by a variety of factors, including age, education, communication between couples about FP, the number of living children, husband approval of FP, the intended number of children, place of residence, religion, knowledge, and attitudes [9–11].

A Bayesian hierarchical modeling study which was conducted by Vladimíra.etal in 2020, demonstrated that currently there are 1.9 billion women in the globe who are of reproductive age [12–46], of whom 1.1 billion require family planning, and the number of women in this age group is predicted to rise. Of them, 842 million utilize contraceptives and 270 million still require contraceptives, which is still not being met [47]. To enhance women’s sexual and reproductive health, non-governmental organizations and the government are now working together in Ethiopia to provide outreach programs that provide contraceptives [48]. Since then, a health sector transformation plan (HSTP) has been formed by the Federal Ministry of Health (FMoH) to raise the contractive prevalence rate. But the demand for modern contraception was unsatisfied [49]. Due to the higher unmet demand and lower usage of modern contraceptives, women are likely experiencing unintended and untimely pregnancies. Unwanted pregnancy has a variety of negative effects on a woman’s health and economic development. For example, it increases maternal mortality, encourages prenatal depression, stunts children’s growth because of malnutrition caused by frequent births, compromises the bond between the mother and child, and lowers women’s participation in the workforce and politics [12–15].

In Ethiopia due to the lack of updated and reliable figures on spatial variation of modern contraceptive, it is difficult to establish policies and programs for the promotion of modern contraceptive use and to take an intervention to decrease unmet need of modern contraceptive use across the region. Therefore, the major goal of this study was to evaluate spatial variations in modern contraceptives use and the contributing factors among women of reproductive age Ethiopia. It is expected to have the following significance.

The document was used to access spatial variations of modern contraceptive use and determinants factors of modern contraceptive use. Therefore, it is important for the stakeholders to understand various factors that affects modern contraceptive use. Additionally, the findings of this study would provide better evidence for policymakers, ministry of health and other stakeholders, which in turn might enable designing and executing appropriate interventions at different levels to increase the rate of modern contraceptive usage, to reduce unmet need of modern contraceptive and to improve the health system as a whole.

## Methods and data source

### Study design, setting and period

The secondary data for this analysis were obtained from mini-Edhs of 2019 that was found at DHS portal of (https://dhsprogram.com/data/dataset/Ethiopia_Interim-DHS_2019.cfm?flag=1). The 2019 EMDHS sample was stratified and selected in two stages. Each region was stratified into urban and rural areas, yielding 21 sampling strata. Samples of EAs were selected independently in each stratum in two stages. To ensure that survey precision was comparable across regions, sample allocation was done through an equal allocation where in 25 EAs were selected from eight regions. However, 35 EAs were selected from each of the three larger regions: Amhara, Oromia, and the Southern Nations, Nationalities, and Peoples’ Region (SNNPR). In the first stage, a total of 305 EAs (93 in urban areas and 212 in rural areas) were selected with probability proportional to EA size (based on the 2019 EPHC frame) and with independent selection in each sampling stratum. A household listing operation was carried out in all selected EAs from January through April 2019. The resulting lists of households served as a sampling frame for the selection of households in the second stage. In the second stage of selection, a fixed number of 30 households per cluster were selected with an equal probability systematic selection from the newly created household listing. All women age 15-49 who were either permanent residents of the selected households or visitors who slept in the household the night before the survey were eligible to be interviewed. In all selected households, women age 15-49 were interviewed using the Woman’s Questionnaire. A total of 9150 households were selected for the sample, of which 8794 were occupied. Of the occupied households, 8663 were successfully interviewed, yielding a response rate of 99%. In the interviewed households, 9012 eligible women were identified for individual interviews; interviews were completed with 8885 women, yielding a response rate of 99%. Overall, there was little variation in response rates according to residence; however, rates were slightly higher in rural than in urban areas. From 8885 interviewed women,5934 were from rural area and the remaining 2951women were from urban area [6]. Since the outcome variable for this study was modern contraceptive utilization .so, the final sample size for this analysis was 8885.

### Study variables

**The outcome variable** for this study was the modern contraceptive use, which was coded as “0” if the women use modern contraceptive and “1” if the women not use modern contraceptive (No method, Folkloric method, Traditional method).

**Individual-level variable:** maternal age, educational status, religion, sex of household, wealth status, current marital status and number of children.

**Community-level variable:** Region and place of residence.

### Inclusion and exclusion criteria

All women who were found within the range of reproductive age groups (15-49 years) included in this study. All women who were outside the range of the reproductive age group (15-49 years) excluded from this analysis.

### Data management and analysis

In all the analyses, we adjusted for the complex nature of the survey design by accounting for clustering, stratification, and weighting. Due to the comparisons and combination (pooled data) of surveys from different regions, with different target population sizes, the weights were denormalized. This was done by dividing the women’s standard weights and their total number the country by the respective survey sampling fraction. Data Extraction, recoding, and both descriptive and analytical analysis were carried out using STATA version 14 software. The multilevel analysis was fitted due to the hierarchical nature of the demographic health survey data. In this study, the multilevel mixed-effects model was employed and the dependent variable was binary.

The Intraclass Correlation Coefficient (ICC) was employed to assess the variability across the region. Bi variable analysis was first done for maternal age, region, place of residence, educational status, religion, sex of household, wealth status, current marital status and number of children, to select variables for multivariable analysis and variables with *p*-value less than 0.05 were considered for multivariable analysis.

### Spatial analysis

In Stata 14, the weighted frequency of modern contraceptives, cluster number, and geographic coordinate data were combined. Data was then exported to Excel and imported into ArcGIS 10.7 for spatial analysis.

### Spatial autocorrelation analysis

The spatial autocorrelation (Global Moran’s I) statistic examines the distribution of modern contraceptives usage among Ethiopian women of reproductive age. Moran’s I is a spatial statistic that uses the entire data set to generate a single output value that varies from − 1 to + 1 in order to evaluate spatial autocorrelation. I, Moran’s Values around − 1 suggest scattered modern contraceptive usage, whereas values near + 1 indicate clustered modern contraceptive use, and values near 0 indicate random distribution of modern contraceptive use. A statistically significant Moran’s I (*p* < 0.05) lead to the failure to reject the alternative hypothesis and rejection of the null hypothesis (modern contraceptive use is randomly distributed) and indicates the presence of spatial autocorrelation.

### Hot spot analysis (Getis-OrdGi* statistic)

The GI* statistics for each area were computed to determine how spatial autocorrelation varies in Ethiopia using Getis-OrdGi* statistics. The *p*-value is estimated for significance using Z-score in order to determine the statistical significance of clustering. High GI* statistical output suggests a “cold area,” whereas low GI* statistical output indicates a “hot spot.”

### Spatial interpolation

To determine the impact of a particular event throughout the country, it is highly expensive and time-consuming to gather trustworthy data. As a result, using the observed data, interpolation was utilized to estimate a portion of a certain area. Based on sampled EAs from DHS, the spatial interpolation approach forecasts modern contraceptive usage in the un-studied portions of the country. In this work, modern contraceptive use in unobserved regions of Ethiopia was predicted using the standard Kriging spatial interpolation approach. The burden of modern contraceptive usage in unsampled regions was estimated for this study using the standard Kriging approach.

### Ethical consideration

The measure DHS program used secondary publically accessible survey data, thus ethical review and participant permission were not required for this particular study. We asked DHS Program for permission to obtain and use the data for this study from their website, and they approved. The National Research Ethics Review Committee (NRERC) of the Ministry of Science and Technology as well as the Ethiopian Health Nutrition and Research Institute (EHNRI) Review Board granted clearance for the EMDHS data collection.

## Result

### Sociodemographic characteristics and bivariate analysis

This study includes a total weighted sample of 8885 reproductive age women from the 2019 mini-Ethiopian demographic and health survey. 2210 (24.9%) of the total study participants were between the age range of 15-19 years, 6024(67.8%) were from rural areas, 3701 (41.7%) were not attending formal education, 3685(41.5%) were orthodox, 7050 (79.3%) were male headed household, 268 (11.9%) were poorest 5743 (64.6%) participants. Were married and 2262(25.5%) use modern contraceptive. Bivariable logistic regression was employed for age, place of residence, religion, educational status, sex of household, wealth index current marital status, among reproductive age women. The result of the bivariable analysis demonstrated that contraceptive use had significant relationships with age, place of residence, religion, educational status, sex of household, wealth index, current marital status among reproductive age women. Variables having a *p*-value less than 0.05 were considered in multivariate analysis. (Table 1).

**Table 1:** Sociodemographic characteristics and Bivariate analysis of factors associated with modern contraceptive use among reproductive age women in Ethiopia 2022. *n* = 8885

List of variables		Frequency	Percentage	COR with 95% CI
**Age**	15-19	2210	24.9	1
	20-24	1481	16.7	1.07 (0.92,1.26)
	25-29	1667	18.8	0.99 (0.86,1.16)
	30-34	1160	13.1	1.22 (1.04,1.45) **
	35-39	1065	12.0	1.03 (0.86,1.23)
	40-44	739	8.3	1.23 (1.01,1.50) *
	45-49	563	6.3	1.17 (0.94,1.45}
**place of residence**	Urban	2861	32.2	1
	Rural	6024	67.8	2.19 (1.95,2.46) ***
**Educational status**	Not educated	3589	40.4	0.64 (0.54, 0.75) ***
	Primary	3701	41.7	0.61 (0.55, 0.68) ***
	Secondary	1088	12.2	0.41 (0.33,0.52) ***
	Higher	507	5.7	1
**Religion**	Orthodox	3685	41.5	1
	Catholic	47	0.5	0.064 (0.02,0 .20) ***
	Protestant	2435	27.4	0.008 (0.004, 0.015) ***
	Muslim	2619	29.5	0.42 (0.37, 0.47) ***
	Traditional	83	0.9	1
	Other	15	0.2	1
**Sex of household head**	Male	7050	79.3	1.15 (1.03,1.28) **
	Female	1835	20.7	1
**Wealth index**	Poorest	1437	16.2	0.71 (0.61,0.83) ***
	Poorer	1615	18.2	0.59 (0.51,0.69) ***
	Middle	1671	18.8	0.59 (0.51,0.70) ***
	Richer	1874	21.1	0.41 (0.35,0.46) ***
	Richest	2287	25.7	1
**current marital status**	Single	2325	26.2	1
	Married	5743	64.6	1.34 (1.18, 1.50) ***
	Living with partner	121	1.4	0.86 (0.52, 1.38)
	Widowed	185	2.1	1.24 (0.89, 1.72)
	Divorced	377	4.2	2.33 (1.87, 2.92) ***
	No longer living together	133	1.5	0.84 (0.56,1.24)
**Current contraceptive use by method type**	No method	6515	73.3	
	Folkloric method	2	0.0	
	Traditional method	105	1.2	
	Modern method	2262	25.5	

*COR* Crude odd ratio, *CI* Confidence interval; statistically significance = *p-value < 0.05; **P < 0.01; ***P < 0.001.

### Spatial analysis results

#### Spatial distribution of modern contraceptive use

**Fig. 1:**
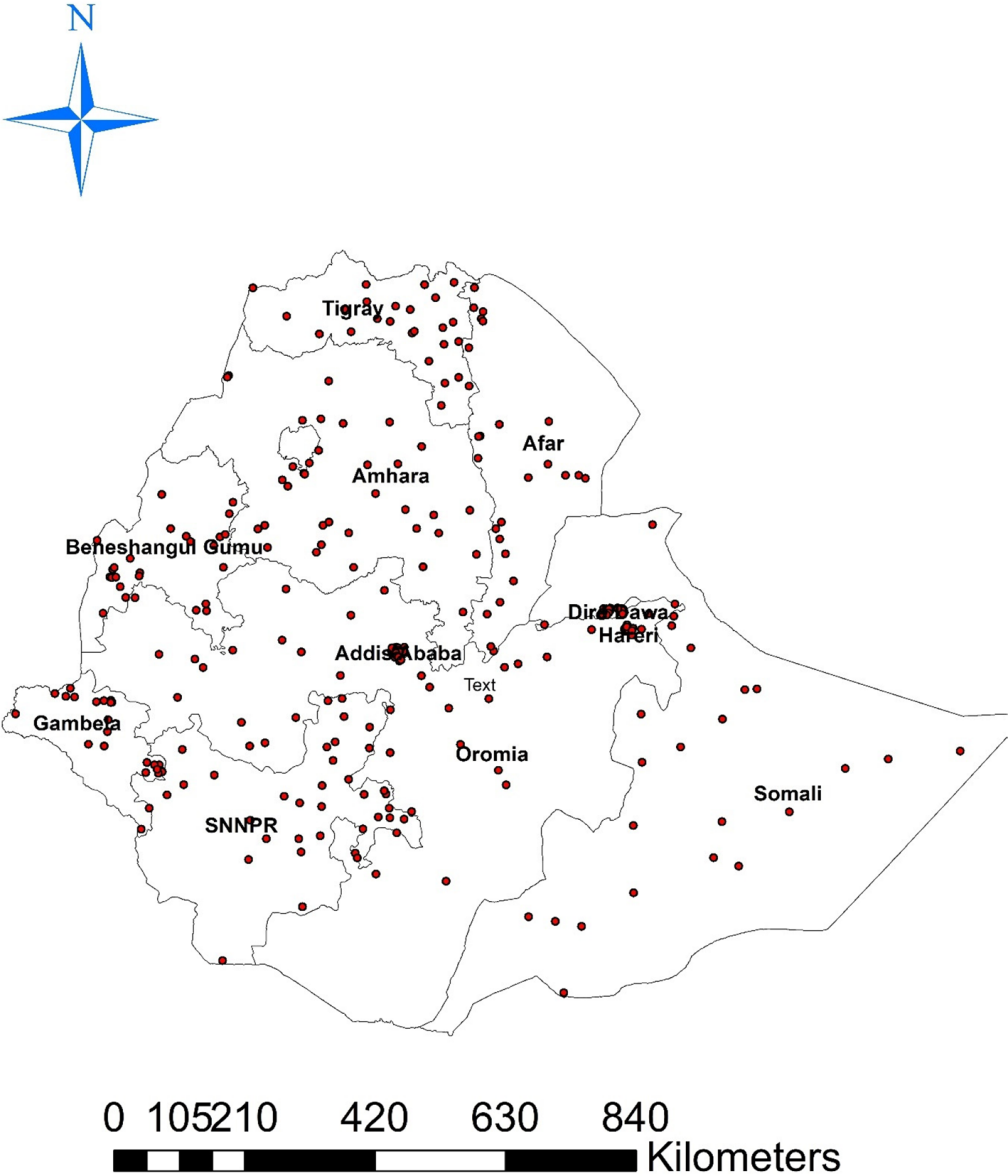
Spatial distribution of modern contraceptive use in Ethiopia 2022. Given the z-score of 31.0717977979, there is a less than 1% likelihood that this clustered pattern could be the result of random chance

In Ethiopia, modern contraception use was analyzed geographically using 305 clusters. The number of current contraceptive usage instances in each cluster corresponds to one enumeration area at each spot on the map. This study’s analysis of the spatial distribution of modern contraception use showed that a higher proportion was used in Ethiopia’s northern region. The southern region of Ethiopia had a low rate of modern contraception usage Fig. 1.

#### Spatial autocorrelation modern contraceptive use

**Fig. 2:**
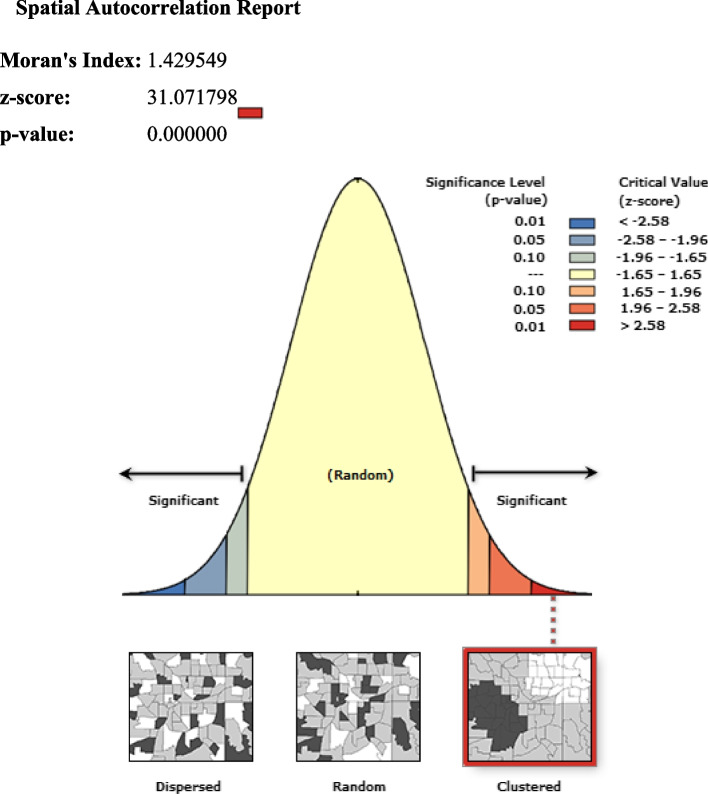
Spatial autocorrelation of modern contractive use in Ethiopia, 2022

The spatial autocorrelation result reveals whether modern contraception use in Ethiopia is randomly distributed across the region, clustered, or dispersed. The results of the spatial autocorrelation study showed a clustering effect in the use of modern contraceptives across the country. The clustered patterns (on the right’s red box side) demonstrated a clustering effect on the usage of modern contraceptives in Ethiopia. The outputs have automatically generated keys on the right and left sides of each panel. The probability that this clustered pattern is the result of random chance is less than 1%, according to the z-score of 31.07 (*p*-value = 0.001). The bright red and blue colors to the end tails indicate an increased level of significances Fig. 2.

#### The hotspot analysis result

**Fig. 3:**
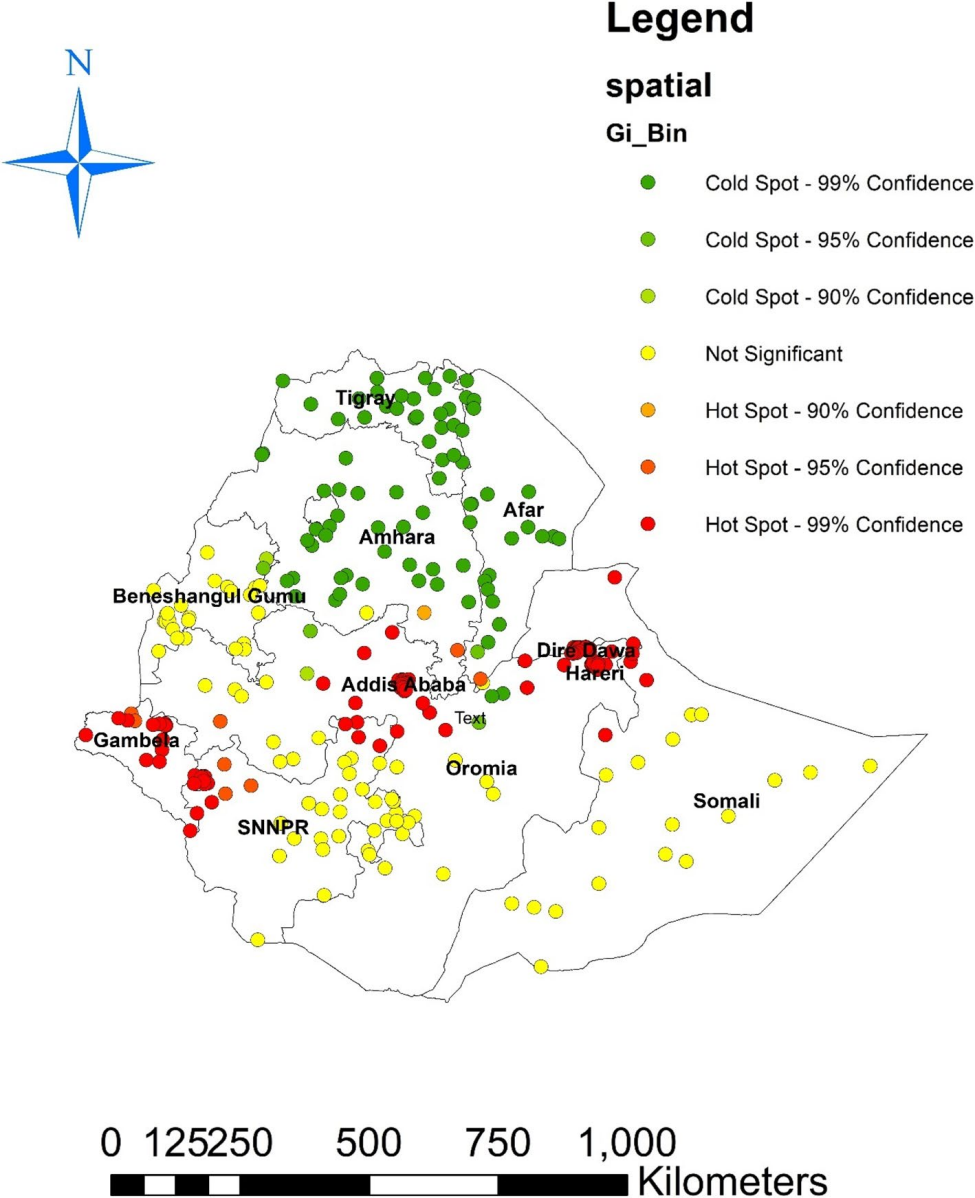
Hotspot analysis of modern contraceptive use in Ethiopia, 2022

The hotspot analysis result shows the low proportion (hotspot) and high proportion (cold spot) areas of modern contraceptives use in Ethiopia. The blue colors were seen in the Tigray, Afar, Amhara, and northern part of Oromia regional states, which are cold spot areas (high proportion of modern contraception use). The red-colored hotspots (areas with a low percentage of women using modern contraceptives) were found in Gambella, SNNPRS. A region of Oromia, Diredawa, and Harari Fig. 3.

#### Spatial interpolation or prediction

**Fig. 4:**
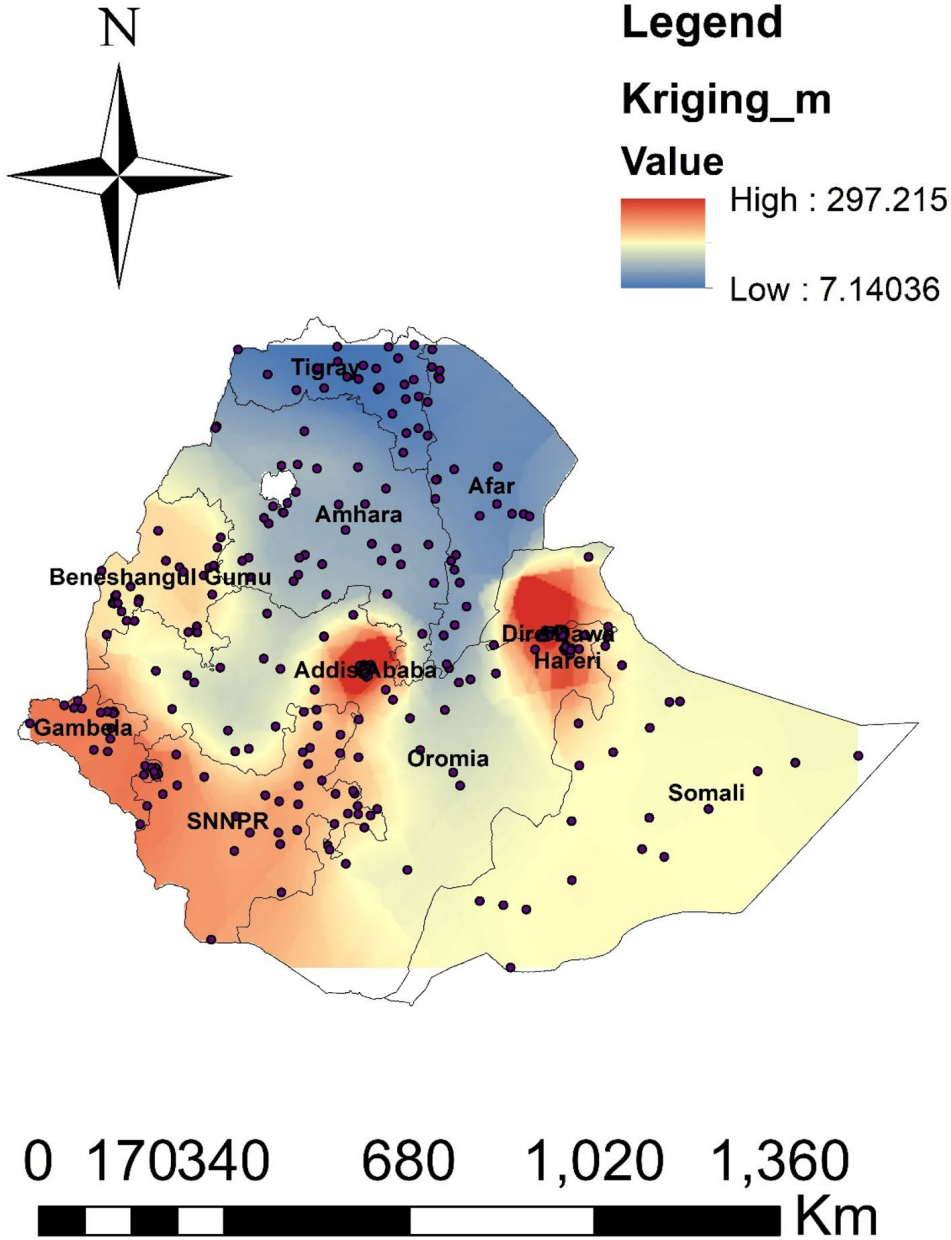
Spatial interpolation of modern contractive use in Ethiopia, 2022

Based on the sampled region, the spatial interpolation approach predicts the proportion of modern contraceptive use for unsampled areas. The area map was described using the standard Kriging method. The red color represents the projected low use of modern contraceptives. If the area’s color shifted from red to blue, it indicates that more people in the area are using modern contraceptives than was previously expected. The country is predicted to utilize modern contraceptives at a high rate, as shown by the blue color. According to the prediction’s results, Tigray, Afar, Amhara, and several areas of Oromia have high rates of modern contraception utilization. The red color prediction showed that the regions of Gambella, Benishangul, SNNPR, Oromia, Somalia, Harari, and Diredawa had the lowest rates of modern contraception usage nationwide Fig. 4.

### Model comparison

Four models were built for this multistage investigation. The first model was built. Without independent factors, it is possible to determine how community variation affects women’s usage of modern contraceptives. The second model included variables at the individual level. Community level characteristics were incorporated in the third model. Finally, the fourth model took into account factors at both the individual and community levels. The ICC in the null model showed that among women of reproductive age, there was a variance in contraceptive usage of 9.97% in the communities. The variance in contraceptive usage among women of reproductive age is described by variables at the individual level in 17.62% of occurrences. The difference in contraceptive usage among women of reproductive age is accounted by 12.35% community level variables. In the end, 19.47% of the variances among women in reproductive age were caused by variables at the individual and community levels. Deviance was used to evaluate model fitness for model comparison (AIC). As a result, it was determined that Model IV, which included factors at both the individual and community levels and had the lowest deviance (AIC) value, provided the best suit. Variables having a *p* < 0.05 significance level were considered to be significant predictors of current modern contraceptive usage among reproductive-age women. Table 2.

**Table 2:** revealed the random effect of modern current contraceptive use and model comparison

Parameters	model I	model II	model III	Model IV
ICC (%)	9.97	17.62	12.35	19.47
Model fitness				
**AIC**	3812	3601	3735	3436

### Pearson Chi-Square analysis of factors associated with modern contraceptive use

The Pearson Chi-square analysis was employed for age, place of residence, religion, educational status, sex of household head, wealth index current marital status, among reproductive age women. The result of the Pearson Chi-square analysis demonstrated that contraceptive use had significant association with place of residence, religion, educational status, sex of household, wealth index, current marital status among reproductive age women. Table 3.

**Table 3:** Pearson Chi-Square analysis of factors associated with modern contraceptive use among reproductive age women in Ethiopia 2022

List of variables	Pearson Chi-Square	DF	P-value
**Age**	11.019^a^	6	0.088
**Type of place of residence**	178.749^a^	1	0.000
**Religion**	985.552^a^	5	0.000
**Educational attainment**	130.781^a^	5	0.000
**Sex of household head**	6.583^a^	1	0.010
**Wealth index**	182.214^a^	4	0.000
**Current marital status**	68.241^a^	5	0.000

*DF* Degree of freedom; statistically significance = *p-value < 0.05; **P < 0.01; ***P < 0.001.

### Multivariable logistic regression

According to the result of the multivariable regression the key variables related with modern contraceptive use among reproductive age women were women’s age, place of residence, educational status, religion, sex of household head, wealth index, current breast feeding and current marital status. The odd of using modern contraceptive among reproductive age women who were live in urban area were two times more likely [AOR = 2.13; 95% CI = (1.75, 2.61); *P* = 0.000] relative to women who were live in the rural area. The odd of modern contraceptive use among reproductive age women who were attending primary education were 0.91 times less likely to use modern contraceptive [AOR = 0.91; 95% CI = (0.74, 1.12); *P* = 0.000] compared to women who were attending higher education. The odd of modern contraceptive use among reproductive age women who were Muslim were 0.25 times less likely to use modern contraceptive [AOR = 0.25; 95% CI = (0.22, 0.29); *P* = 0.000] relative to women who were orthodox. The odd of contraceptive use among male headed household were 1.49 times more likely to use modern contraceptive [AOR = 1.49; 95% CI = (1.29, 1.70); *P* = 0.000] compared to female headed household. The odd of modern contraceptive use among poorest were 0.54 times less likely to use contraceptive [AOR = 0.54; 95% CI = (0.45, 0.66); *P* = 0.000] relative to women who were richest. The odd of modern contraceptive among married were 1.42 time more likely to use contraceptive [AOR = 1.42; 95% CI = (1.19, 1.70); P = 0.000] relative to women who were single (Table 4).

**Table 4:** Multivariable analysis of factors associated with modern contraceptive use among reproductive age women in Ethiopia 2022. (*n* = 8885)

Variables	Categories	Modern contraceptive		AOR with 95% CI
		Yes	No	
Age	15-19	461	1639	1
	20-24	367	1211	1.03 (0.85, 1.23)
	25-29	384	1368	0.84 (0.68, 1.03)
	30-34	299	867	0.91 (0.72, 1.14)
	35-39	233	804	0.68 (0.53, 0.86) **
	40-44	184	530	0.84 (0.65,1.09)
	45-49	133	405	0.77 (0.58, 1.02)
place of residence	Rural	1627	4307	1
	Urban	434	2517	2.13 (1.75, 2.61) ***
Educational status	Not educated	1050	2590	0.91 (0.74, 1.12)
	Primary	666	2679	0.80 (0.69,0.93) **
	Secondary	237	912	0.64 (0.49, 0.84) ***
	Higher	107	643	1
Religion	Orthodox	1294	2080	1
	Catholic	3	75	0.04 (0 .01, 0.14) ***
	Protestant	8	1703	0.005 (0.003, 0.011) ***
	Muslim	756	2879	0.25 (0.22, 0.29) ***
	Traditional	0	60	1
	Other	0	27	1
Sex of household head	Male	1434	4946	1.49 (1.29, 1.70) ***
	Female	1878	627	1
	Poorest	662	1878	0.54 (0.45, 0.66) ***
Wealth index	Poorer	301	1040	0.48 (0.39, 0.58) ***
	Middle	324	944	0.46 (0.38, 0.55) ***
	Richer	300	1044	0.39 (0.31, 0.49) ***
	Richest	474	2427	1
current marital status	Single	444	1856	1
	Married	1359	4254	1.42 (1.19, 1.70) ***
	Living with partner	22	107	0.81 (0.48,1.36)
	Widowed	52	175	1.36 (0.90, 2.04)
	Divorced	152	272	1.99 (1.51, 2.62) ***
	No longer living together	32	160	0.83 (0.53, 1.30)

*AOR* Adjusted odd ratio, *CI* Confidence interval; statistically significance = *p-value < 0.05; **P < 0.01; ***P < 0.001.

## Discussion

The study assessed the spatial distribution of modern contraceptive use and the factors that influence its use among women of reproductive age in Ethiopia, using the recent Ethiopian mini demographic health survey data conducted in 2019. In general, this study showed geographical variations in the usage of modern contraceptives among women of reproductive age. In Ethiopia, the northern region had a higher proportion of women using modern contraceptives, while the southern region had a lower proportion. Particularly, areas of southern Ethiopia including Gambella, Benishangul, SNNPR, Oromia, Somalia, Harari, and Diredawa had low rates of modern contraception use. Furthermore, the northern region of Ethiopia had the highest rate of modern contraceptive use. High rates of modern contraception use are found in Tigray, Afar, Amhara, and several regions of Oromia. This variations were existed, because of Ethiopia is a low-income country with limited access to family planning (FP), particularly in the developing regions [16]. The Ethiopian government has significantly increased the number of medical facilities and qualified personnel [17]. In turn, this led to a rise in the country’s contraceptive prevalence rate (CPR), which increased from 8% in 2000 to 41.4% in 2019 [18]. However, there was a significant regional variation in CPR among the developing regions [19]. Additionally, we hypothesize that this discrepancy may result from regional socioeconomic disparities.

The results of the bivariable analysis in this study showed that among women of reproductive age, the use of contraception was significantly correlated with maternal age, place of residence, religion, educational status, sex of household, wealth index, and current marital status. According to other similar studies that supported this finding, the use of modern contraceptives is influenced by a variety of factors, including age, education, communication between couples about FP, the number of living children, husband approval of FP, the intended number of children, place of residence, religion, knowledge, and attitudes [9–11].

This survey revealed that 25.5% of women of reproductive age used modern contraceptives. This finding was slightly higher than the studies which was conducted in Ethiopia [20] and in Ghana [21]. On the other hand the finding of this study was lower than the study which was conducted in north west Ethiopia [22] Kenya [23] and in Ethiopia [24]. These variations may have been caused by the study period, sample size, and study area’s locations.

The findings of this study indicated that women of reproductive age who resided in urban areas had higher odds of using modern contraceptives than did women who lived in rural areas. This finding was supported by the studies which was conducted in Nigeria [25] and in Senegal [26]. This discrepancy may have existed due to the great availability of family planning services and the rising number of health institutions in urban areas. However, most women face several barriers to obtaining and using modern contraceptives, particularly those who live in rural areas [27] such as, low educational attainment in rural area [28], Poverty rates in rural areas have been consistently higher than those in urban area [29], deep rooted cultural belief [30] especially, the husband’s role as primary decision-maker and the desire for a large family [31], fear of side-effects due to lack of knowledge [32], long distances to healthcare facilities, and inadequate stock of preferred types of modern contraceptives [33, 34].

According to this study, women of reproductive age who were attended in primary education were less likely to use modern contraceptives than those attended in higher education. This finding was consistent with the studies which was conducted in Ethiopia [35], in Kenya [36] in Zaire [37]. The first possible reason for these variation were women who receive more maternal education may be more informed about the variety of contraceptive methods available, which will enable them to use contraception more effectively and make informed decisions [38]. The second reason were, when education levels rise, wealth and prestige tend to rise as well, and the desire to limit family size by utilizing modern contraceptives would increase [39].

The results of this study showed that Muslim women in reproductive age used modern contraceptives at a lower rate than orthodox and protestant women. This was in line with the studies which was conducted in Ethiopia [40, 41], Zambia [42], Ghana [21] and Tanzania [43]. Religious acceptance of family planning methods might be one reason for this variation, although participants’ interpretations of their religion’s position on the topic varied. Most people who believed that family planning was incompatible with their beliefs declared that they had a duty to have as many children as God would allow them to have. Others thought family planning was appropriate given their moral obligation to raise and safeguard their children by reducing the number of children [43]. Additionally, there are misinterpretations of Islamic teachings on polygamy, which is still practiced and has a negative impact on FP adoption, as well as contraceptives, which is frequently prohibited [44].

This study demonstrated that male headed households were more likely than female headed households to utilize contraceptives. This study finding was concurrent with studies which was conducted in Ethiopia [45], in Tanzania [46]. Since, Interspousal communication is a key issue that affects the sustained use of family planning [50]. Men were viewed as the only ones who could provide the demands of their families. Women were not thought of as decision makers, but rather as implementers of what males had decided, without challenging those decisions [51].

According to this study, women who were poorer were less likely than women who were wealthy to utilize modern contraceptives. This finding was in line with the studies which was conducted in Nigeria [52], in Ethiopia [53] and in Amhara region of Ethiopia [54]. The contributing factors for these disparity were the usage of contraceptives has a financial cost associated with it, rich women might be able to avoid any financial barriers to using modern contraceptives, while poor women might not [55, 56], the level of household wealth has a significant impact on access to education, basic healthcare services, and health information [57].

This study demonstrated that married women were more likely than single women to utilize modern contraceptives. This finding were consistent with studies which were conducted in southern Ethiopia [58], north west Tanzania [59], and Kenya [60]. From the perspective of males as a potential factor that influences the usage of modern contraceptives for the purposes of fertility control, these differences may have arisen [61].

## Conclusion

In this study Individual and community level factors were associated with modern contraceptive use and also there were spatial variations in modern contraceptive use across the region among reproductive-age women. Empowering women to have better educational status, improving the wealth index, promoting marriage, creating awareness among rural residences women and promoting education about modern contraceptives through religiously acceptable persons, and promoting modern contraceptive use in developing regions were the key factors to improve modern contraceptive use among reproductive age women in Ethiopia.

### Strengths and limitations of this study

- The DHS has a similar design, with identical variables in a different environment; the result may, therefore, be applicable to other similar locations.
- The study used a sufficiently large sample size at the national level to ensure its representativeness.
- Recall bias is one of the potential drawbacks, especially for retrospective data based on past experiences.
- The magnitude of the bias is often unknown and correcting for the bias is difficult.
- Since, this study was cross sectional study.it doesn’t showed temporal relationships between independent and dependent variable.

## Data Availability

The data were obtained from mini-Edhs of 2019 that was found at DHS portal of (https://dhsprogram.com/data/dataset/Ethiopia_Interim-DHS_2019.cfm?flag=1).
